# Growth Inhibition, Mortality Induction, Adverse Impacts of Development, and Underlying Molecular Mechanisms of Thymol Against *Spodoptera frugiperda*

**DOI:** 10.3390/insects17010069

**Published:** 2026-01-06

**Authors:** Huiyin Hu, Huanqian Yao, Shuyin He, Xinyi Xie, Cuiting Liu, Veeran Sethuraman, Jingjing Zhang, Benshui Shu

**Affiliations:** 1 Guangzhou City Key Laboratory of Subtropical Fruit Trees Outbreak Control, Zhongkai University of Agriculture and Engineering, Guangzhou 510225, China; 2 Department of Orthopaedics, Saveetha Medical College and Hospital, Saveetha Institute of Medical and Technical Sciences (SIMATS), Chennai 602105, Tamil Nadu, India; sethuramanbio@gmail.com

**Keywords:** *Spodoptera frugiperda*, thymol, growth inhibition, mortality, RNA-Seq, differentially expressed genes

## Abstract

The global migratory pest, *Spodoptera frugiperda*, has garnered widespread attention due to the serious damage it inflicts on agricultural productivity, particularly in maize. Thymol is a phytochemical that exhibits functional diversification in plant defense, encompassing antibacterial activities and insect pest management. However, the impact of thymol on *S. frugiperda* is still undetermined. This study examined the growth inhibition and mortality induction in *S. frugiperda* larvae after thymol exposure. The detrimental effects of 2.0 and 4.0 mg/g thymol treatments on the growth and development of *S. frugiperda* were also examined. RNA-Seq was used to investigate the probable toxicological mechanism of thymol on *S. frugiperda*, resulting in the identification of 1754 and 1022 DEGs impacted by 2.0 and 4.0 mg/g thymol treatments, respectively. The DEGs associated with chitin metabolism and cuticle synthesis, hormone biosynthesis, and protein and fat digestion were subjected to additional analysis. Our findings demonstrate the efficacy of thymol in controlling *S. frugiperda* and lay the groundwork for understanding the molecular toxicological mechanisms of thymol on larvae.

## Simple Summary

The fall armyworm, *Spodoptera frugiperda*, is an economically important pest that has caused extensive damage to many crops all around the world over the past decade. Due to the advantages of being bioactive, biodegradable, and ecologically safe, thymol, one of the principal ingredients identified in essential oils, has exhibited its potential in pest control. However, the effects and toxicological mechanisms of thymol on *S. frugiperda* are still unclear. In this study, we evaluated the bioactivity of thymol on the larvae of *S. frugiperda*. RNA-Seq was performed to explain the preliminary toxicological mechanisms of thymol on larvae by the identification and functional enrichment analyses of differentially expressed genes (DEGs). Overall, our findings indicate that thymol also exhibits the potential for the control of *S. frugiperda*.

## 1. Introduction

The fall armyworm, *Spodoptera frugiperda* (J.E. Smith) (Lepidoptera: Noctuidae), is a serious economic pest that is native to tropical and subtropical areas of America [1]. First identified in West Africa in 2016, it is now recognized by FAO as a significant invasive pest, affecting over 100 countries globally [2,3]. This pest exhibits high polyphagy. Its host plants include 353 plant species from 76 different families, such as maize, sorghum, rice, soybean, cotton, and peanuts [4]. Maize is the preferred host of *S. frugiperda* [5]. Infections that occur during the crop’s early stages can cause production losses of up to 70% [5]. This pest initially infiltrated China in 2018 [5]. It has now replaced the corn borer *Ostrinia furnacalis* as the predominant pest on maize in the southwestern border region of China [5,6]. Chemical pesticides represent the most traditional management approach for controlling *S. frugiperda*, owing to their high efficiency, rapid action, and satisfying outcomes [7]. Nonetheless, *S. frugiperda* has significant adaptability, and resistance to chemical pesticides has developed in several geographic populations [8,9]. Therefore, it is essential to investigate viable strategies for pest management.

The connections between plants and herbivorous insects are intricate [10]. Throughout prolonged co-evolution, plants have evolved a diverse array of allelochemicals to defend themselves against herbivorous insects [10]. Essential oils (EOs) are primary constituents of plant allelochemicals, comprising numerous monoterpenes, diterpenes, sesquiterpenes, and other aromatic compounds [11,12]. They serve as defensive chemicals against herbivorous insects through many mechanisms. These include insecticidal, anti-oviposition, repellent, and antifeedant properties [11]. Moreover, EOs are regarded as being viable alternatives to chemical pesticides in pest management. They are effective at low concentrations; non-toxic to mammals, birds, and fish; rapidly break down in soil; are convenient to administer; and are slow to develop pest resistance [12]. Distinct insecticidal effects of EOs from 57 plant species against *S. frugiperda* were noted [13]. Among them, the EOs from *Ageratum conyzoides*, *Piper septuplinervium*, *Ocimum gratissimum*, and *Siparuna guianensis* were recognized as being the most efficacious [13].

Thymol (2-isopropyl-5-methylphenol) is a prominent monoterpenoid phenol. It is the principal ingredient of essential oils from several Lamiaceae plants, including thyme (*Thymus vulgaris* L.) and oregano (*Origanum vulgare* L.) [14,15]. It is extensively utilized as a secure bioactive substance for food packaging, owing to its antibacterial characteristics and non-toxicity to vertebrates and the natural ecosystem [14,16]. Beyond this application, thymol also exhibits multiple biological effects against various insects. The significant repellency effects and death of *Sitophylus oryzae* were elicited by varying the concentrations of thymol treatments (2%, 4%, and 6%) (*w*/*w*) [17]. Furthermore, thymol influenced *Helicoverpa armigera* in many manners, including oviposition suppression, growth retardation, and induction of mortality [18]. Moreover, thymol has shown antifeedant properties against *Rhynchophorus ferrugineus* larvae [19]. Thymol exhibited significant toxicity against third-instar *S. frugiperda* larvae, with a 48 h LD_50_ of 4.91 mg/g [20]. However, the mechanisms of thymol against *S. frugiperda* remain unknown.

In this study, the impact of different thymol concentrations on the survival and growth of *S. frugiperda* larvae was investigated. Additionally, the adverse effects of 2.0 and 4.0 mg/g thymol exposure on the development of *S. frugiperda* were documented. Comparative transcriptomics were conducted to investigate the molecular mechanisms of thymol against *S. frugiperda* larvae. The DEGs affected by thymol exposure were identified. Key DEGs associated with chitin metabolism and cuticle synthesis, hormone biosynthesis, protein and fat digestion, and immunological response were further analyzed. The alterations in mRNA expression levels of several DEGs influenced by thymol treatments were subsequently validated via RT-qPCR. The results established a basis for additional toxicological mechanism investigations of thymol against pests and validated its prospective application for controlling *S. frugiperda* in the field.

## 2. Materials and Methods

### 2.1. Insect Rearing

The laboratory population of *S. frugiperda* originated from individuals gathered in the cornfields of Conghua District, Guangzhou City, Guangdong Province, China. The population was sustained without exposure to insecticides for over four years. The larvae were fed with an artificial diet, while the adults were fed with a 5–10% honey water solution. The artificial diet, weighing 1.0 kg, comprised 100 g of cornflour, 80 g of soy flour, 26 g of yeast powder, 26 g of agar, 8.0 g of vitamin C, 2.0 g of sorbic acid, 1.0 g of choline chloride, 0.2 g of inositol, and 0.2 g of cholesterol [21]. The larvae were reared in plastic boxes. The adults were housed in paper tubes (diameter 10 cm, height 20 cm). They were kept in an incubator under the conditions of 25 ± 1 °C, 70% relative humidity, and a 12:12 h light–dark cycle.

### 2.2. Bioassay of Thymol Against S. frugiperda Larvae

Thymol, with 98% purity, was purchased from Aladdin (T104425, Shanghai Aladdin Bio-Chem Technology Co., Ltd., Shanghai, China). It was dissolved with dimethyl sulfoxide (DMSO, #D8372, Beijing Solarbio Science & Technology Co., Ltd., Beijing, China). Equal amounts of various thymol stock solutions were included in artificial diets to achieve the final concentrations of 0.50, 1.0, 2.0, 3.0, and 4.0 mg/g, respectively. The newly molted third-instar larvae were collected for a bioassay. The weight of twenty larvae was controlled at about 0.075 g. To prevent cannibalism, the individual was maintained in the *Drosophila* vial separately. Then, the artificial diets with different concentrations of thymol were placed into the *Drosophila* vial, respectively. Three biological replicates were conducted for each treatment. Twenty individual larvae with the same treatment constituted one replicate. The larvae, fed with an artificial diet combined with DMSO, served as controls. The unconsumed artificial diet in the vial was removed and replaced with a fresh batch of the corresponding diet every day. The larval weight of the samples subjected to various treatments for 1 and 3 days was measured by using an electronic balance (Sartorius, Göttingen, Germany) with a precision of one ten-thousandth. Additionally, the larval mortality across several samples was quantified.

### 2.3. Larval Feeding Trials

The newly molted third-instar larvae were collected for the feeding trials. The artificial diets were created with final thymol concentrations of 2.0 mg/g and 4.0 mg/g, respectively. One hundred and eighty caterpillars (n = 60 per group) were individually inserted into the sterile *Drosophila* vials to prevent cannibalism. Subsequently, suitable artificial diets with various treatments were introduced into the vials accordingly. Twenty larvae fed with an identical diet constituted a single sample, but development was followed individually. Three biological replicates were conducted for each treatment. The larvae that were fed with the diet containing DMSO served as the control samples. The diet in the vial was refreshed daily. Following three days of feeding, the diet in all vials was substituted with an artificial diet that was devoid of any additives until pupation occurred. The period of larval development and the pupation rate across several groups were subsequently documented. The pupa was subsequently weighed and preserved under identical conditions until eclosion. Subsequently, the eclosion rates of various groups, adult weight, and adult lifespan were recorded.

### 2.4. RNA Isolation and Transcriptome Sequencing

To investigate the molecular mechanisms of thymol against *S. frugiperda*, the surviving larvae that were subjected to 0, 2.0, and 4.0 mg/g thymol treatments for 3 days were collected for RNA sequencing. Ten survival larvae with the same treatments were used as one sample. Three biological replicates were collected for each treatment. The samples were placed in a mortar, followed by the addition of liquid nitrogen, respectively. Following pulverization, the total RNA from nine samples subjected to three treatments was extracted using TRIzol reagent (Invitrogen, Carlsbad, CA, USA), in accordance with the manufacturer’s instructions. The integrity and concentration of the total RNA were assessed using the Agilent 2100 Bioanalyzer (Agilent Technologies, Palo Alto, CA, USA) and the NanoDrop 2000 (Thermo Fisher Scientific, Waltham, MA, USA). The quality-assured RNA was utilized for cDNA library creation.

The synthesis of the cDNA library and RNA sequencing was conducted on the platform of Wekemo Tech Co., Ltd., located in Shenzhen, China. One μg of total RNA from each sample was utilized for the cDNA library creation, using the NEBNext^®^ Ultra^TM^ RNA Library Prep Kit (NEB, Beijing, China). mRNA was isolated from total RNA utilizing poly-T oligo-conjugated magnetic beads. Subsequently, they were fragmented with divalent cations in NEBNext First Strand Synthesis Reaction Buffer at elevated temperatures. The fragments were subsequently reverse-transcribed into first-strand cDNA with a random hexamer primer and M-MuLV reverse transcriptase (RNase H-). The second-strand cDNA was synthesized using DNA polymerase I and RNase H. Following end-repair, phosphorylation, and the addition of ‘A’ bases, cDNA fragments measuring approximately 250–300 bp were purified using the AMPure XP system (Beckman Coulter, Beverly, MA, USA). Then, they were amplified using Phusion High-Fidelity DNA Polymerase. The PCR products were subsequently purified using AMPure XP technology. Subsequent to the successful completion of a quality inspection, the cDNA libraries were sequenced, utilizing an Illumina platform.

### 2.5. De Novo Assembly and Differentially Expressed Genes Identification

The raw data underwent quality control, resulting in clean reads following the elimination of adaptor sequences, poly-N regions, and low-quality reads. The Q20, Q30, and GC content of the clean reads across several samples were computed. The clean reads were subsequently aligned to the reference genome of *S. frugiperda* (GenBank ID: GCF_023101765.2, https://www.ncbi.nlm.nih.gov/datasets/genome/GCF_023101765.2/, accessed on 26 August 2022), utilizing Hisat2 v2.0.5. The mapped reads were constructed using StringTie (v1.3.3b) through a reference-based method. Additionally, StringTie software was also used for quantifying gene expression levels with the calculation method of the transcripts per million reads (TPM) methodology. Thymol treatment resulted in the identification of differentially expressed genes (DEGs) by DESeq2, based on the criteria of |log2Fold Change| ≥ 1 and an adjusted *p*-value < 0.05. Additionally, Gene Ontology (GO) and Kyoto Encyclopedia of Genes and Genomes (KEGG) enrichment analyses of DEGs were conducted, using the ClusterProfiler R package 4.0.5. The GO terms and KEGG pathways that were significantly enriched with DEGs were assessed with a corrected *p* value of less than 0.05.

### 2.6. Real-Time Quantitative PCR

RT-qPCR was used to verify the precision of transcriptome sequencing outcomes. Sixteen DEGs influenced by thymol treatments were chosen as the target genes. The primers for the target and reference genes were constructed utilizing Primer 5.0 software, as presented in Supplemental Table S1. The HiScript^®^ III All-in-one RT SuperMix Perfect for qPCR kit (Vazyme, Nanjing, China) was utilized for cDNA synthesis following the manufacturer’s recommendations. A 10 μL PCR reaction solution was formulated, comprising 5.0 μL of Trans Start^®^ Tip Green qPCR SuperMix (TransGen Biotech, Beijing, China), 0.5 μL of forward primer, 0.5 μL of reverse primer, 1.0 μL of cDNA, and 3.0 μL of ddH_2_O. The reaction solution was subsequently injected onto 384-well plates and amplified using a LightCycler 480 II machine (Roche, Basel, Switzerland). The amplification procedure was listed as follows: one cycle at 95 °C for 2 min, 45 cycles of 95 °C for 20 s, 60 °C for 15 s, and 72 °C for 15 s, and it ended with a melting-curve step. The expression levels of the target genes were assessed using the 2^−△△CT^ technique, with normalization achieved using two reference genes: *Sf-EF1α* (U20139.1) and *Sf-RPL13* (AF400183.1).

## 3. Results

### 3.1. Thymol Induced Mortality and Growth Inhibition in the S. frugiperda Larvae

**Figure 1:**
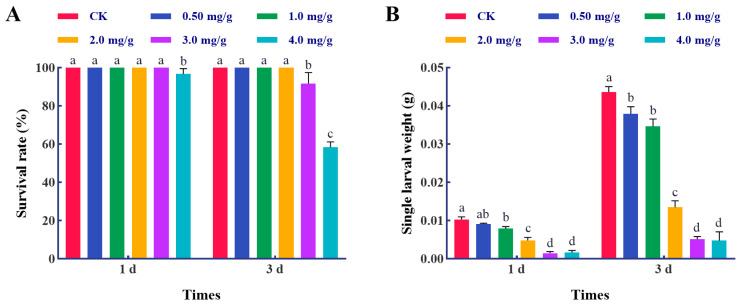
Thymol treatments induced mortality and growth inhibition in third-instar larvae of *S. frugiperda*. (**A**) The survival rates of *S. frugiperda* larvae following treatment with various doses of thymol for 1 and 3 days. (**B**) The individual larval weight of larvae exposed to varying doses of thymol for 1 and 3 days. CK: The larvae were fed with a diet supplemented with DMSO; 0.50, 1.0, 2.0, 3.0, and 4.0 mg/g, which corresponded to the larvae that were subjected to the diet containing 0.50, 1.0, 2.0, 3.0, and 4.0 mg/g thymol, respectively. A one-way ANOVA followed by a Tukey Honestly Significant Difference (HSD) test (*p* < 0.05) was utilized for statistical analysis. Distinct letters above the bars denote groups exhibiting substantial differences.

In this study, the effects of thymol exposures against the larvae of *S. frugiperda* were analyzed. A 3.33% mortality was observed in the larvae with 4.0 mg/g thymol treatment for 1 d, with a significant difference when compared to other groups (*F* = 4.0, *p* = 0.0228) (Figure 1A). The mortality of the larvae subjected to 3.0 mg/g thymol treatment for 3 days was 6.67%, which escalated to 41.67% following 3 days of 4.0 mg/g thymol treatment (*F* = 120.0, *p* < 0.0001). Furthermore, thymol exposure decreased the larval weight after 1 day (*F* = 143.7, *p* < 0.0001) and 3 days of treatments (*F* = 306.2, *p* < 0.0001) (Figure 1B). These data indicate that thymol exerts a harmful effect on the larvae by increasing mortality and inhibiting growth.

### 3.2. Thymol Exposures Altered the Development of S. frugiperda

**Figure 2:**
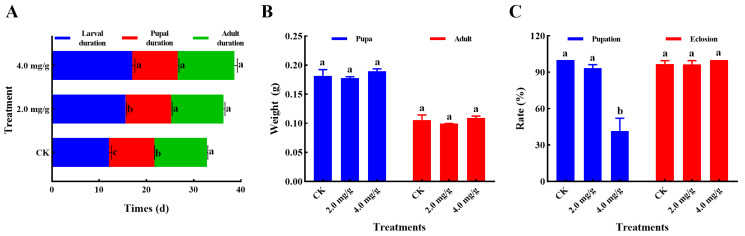
Thymol treatments elicited adverse effects on the development of *S. frugiperda*. (**A**) The impact of 2.0 and 4.0 mg/g thymol exposure on the durations of larval, pupal, and adult stages (n ≈ 20 per sample, three samples per group). (**B**) The impact of 2.0 and 4.0 mg/g thymol exposure on the weight of pupae and adults (n ≈ 20 per sample, three samples per group). (**C**) The impact of 2.0 and 4.0 mg/g thymol exposure on pupation and eclosion rates (n = 3). CK: The larvae were fed with a diet supplemented with DMSO; 2.0 and 4.0 mg/g, which corresponded to the larvae that were administered a diet containing 2.0 and 4.0 mg/g thymol, respectively. A one-way ANOVA, followed by an HSD test (*p* < 0.05), was utilized for statistical analysis. Distinct letters above the bars denote groups exhibiting substantial differences.

In this study, the impact of thymol exposures on the development of *S. frugiperda* was investigated. As shown in Figure 2A, the larval developmental period was markedly extended after treatment with 2.0 and 4.0 mg/g thymol (*F* = 179.1, *p* < 0.0001). A significant difference was seen in the pupal duration in thymol treatment groups when compared to the control group (*F* = 10.09, *p* = 0.0120). No significant difference was seen in the adult duration across different groups (*F* = 0.5985, *p* = 0.5794). Furthermore, no significant variation was observed in the pupal weight (*F* = 2.415, *p* = 0.1701) and adult weight (*F* = 2.462, *p* = 0.1657) among the various groups (Figure 2B). Moreover, a substantial reduction in pupation rate was seen in the group that was administered 4.0 mg/g thymol in comparison to the other groups (*F* = 78.64, *p* < 0.0001). No notable difference was detected in the emergence rates of *S. frugiperda* among the various groups (*F* = 2.005, *p* = 0.2154) (Figure 2C). These data indicate that high concentrations of thymol could adversely affect multiple life history traits of *S. frugiperda*.

### 3.3. Transcriptome Analyses

In this study, transcriptome analysis was employed to investigate the toxicological processes of thymol on the larvae of *S. frugiperda*. RNA-Seq was conducted on samples from controls (n = 3), those treated with 2.0 mg/g thymol (n = 3), and those treated with 4.0 mg/g thymol (n = 3). Following sequencing and quality control, each sample yielded over 40.22 million clean reads. The sequencing quality was excellent, with Q20 and Q30 values exceeding 0.988 and 0.962, respectively (Supplemental Table S2). Over 39.19 million clean reads from each transcriptome were aligned to the reference genome of *S. frugiperda*, with an over-mapping rate above 78.0%. A total of 15,157 genes with expression levels were identified, based on the calculated TPM values.

### 3.4. Identification and Functional Annotation Analysis of Differentially Expressed Genes (DEGs)

**Figure 3:**
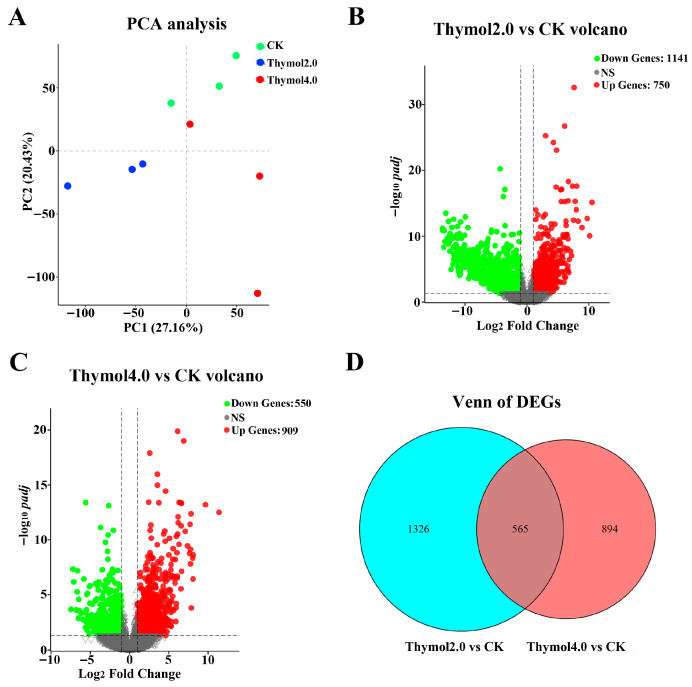
Identification of DEGs in larvae exposed to 2.0 and 4.0 mg/g thymol. (**A**) The PCA analysis of all samples subjected to various treatments. The percentage of variance explained by each principal component is indicated on the axes. (**B**) The Volcano plot of DEGs found in the group subjected to 2.0 mg/g thymol treatment compared to the control group. Red and green marks indicated the up-regulated and down-regulated DEGs, respectively. (**C**) The Volcano plot of DEGs discovered in the group receiving 4.0 mg/g thymol treatment compared to the control group. Red and green marks indicated the up-regulated and down-regulated DEGs, respectively. (**D**) The Venn diagram of DEGs influenced by 2.0 and 4.0 mg/g thymol intake. CK: The larvae were fed on a diet supplemented with DMSO; Thymol 2.0 and Thymol 4.0 corresponded to the larvae that were administered diets containing 2.0 mg/g and 4.0 mg/g of thymol, respectively.

PCA analysis of the nine samples revealed that samples from the same group grouped together, whereas separate groups were clearly separated (Figure 3A). These results indicate that thymol treatments considerably influenced the samples. A total of 1891 DEGs were discovered in the comparison between the 2.0 mg/g thymol treatment group and the control group. Among them, 750 DEGs were up-regulated, whereas 1041 DEGs exhibited down-regulated expressions (Figure 3B). In the group treated with 4.0 mg/g thymol, 1459 DEGs were identified compared to the control group, comprising 909 up-regulated and 550 down-regulated genes (Figure 3C). A total of 565 DEGs were commonly influenced by varying levels of thymol exposure (Figure 3D).

**Figure 4:**
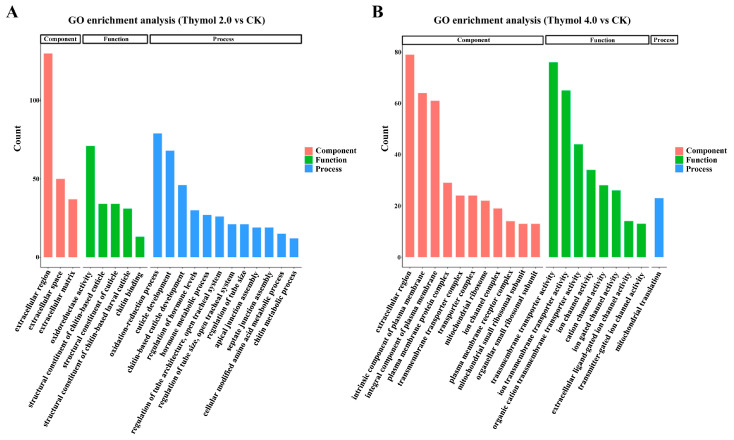
Gene Ontology enrichment analysis of DEGs in larvae exposed to 2.0 and 4.0 mg/g thymol. (**A**) The top twenty significantly enriched GO terms associated with DEGs affected by 2.0 mg/g thymol exposure. The enriched GO terms were categorized into three classifications: biological process, cellular component, and molecular function. (**B**) The top twenty significantly enriched GO terms associated with DEGs affected by 4.0 mg/g thymol exposure. The enriched GO terms were categorized into three classifications: biological process, cellular component, and molecular function. CK: The larvae were fed on a diet supplemented with DMSO; Thymol 2.0 and Thymol 4.0 corresponded to the larvae that were administered diets containing 2.0 mg/g and 4.0 mg/g of thymol, respectively.

The functional annotation of DEGs influenced by thymol treatments was further examined by GO and KEGG enrichment analyses. For larvae exposed to 2.0 mg/g thymol, a total of 738 DEGs were assigned to three main GO categories: biological process (BP), molecular function (MF), and cellular component (CC). Among these, 89 GO terms were significantly enriched (*p*.adjust < 0.05), including 58 BP terms, 7 CC terms, and 24 MF terms. The most enriched BP terms were ‘oxidation–reduction process’ (79 DEGs), ‘cuticle formation’ (65 DEGs), and ‘lipid metabolic process’ (60 DEGs). In the MF category, ‘structural constituent of chitin-based cuticle’ and ‘structural constituent of cuticle’ were assigned the highest number of DEGs. For larvae exposed to 4.0 mg/g thymol, a total of 592 DEGs were enriched in 134 GO terms: 53 BP terms, 29 CC terms, and 52 MF terms. The most notable BP terms were ‘transmembrane transport’ (77 DEGs) and ‘ion transport’ (70 DEGs). Correspondingly, the most prominent MF terms were ‘transporter activity’ (80 DEGs) and ‘transmembrane transporter activity’ (76 DEGs). The top 20 significantly enriched GO terms with the smallest *p*.adjust values influenced by exposure to 2.0 and 4.0 mg/g thymol were presented (Figure 4).

**Figure 5:**
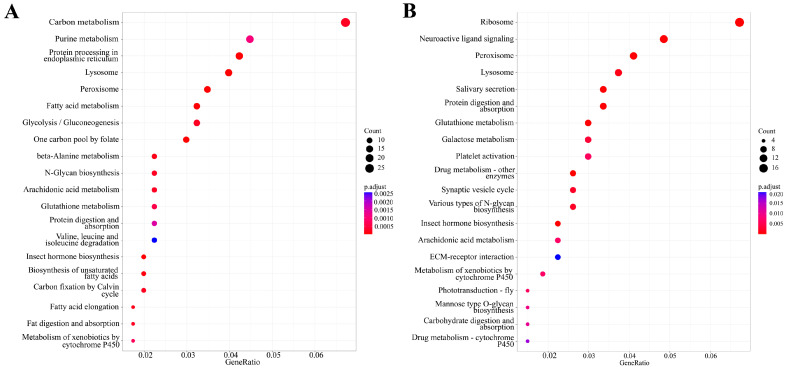
KEGG enrichment analysis of DEGs in larvae exposed to 2.0 and 4.0 mg/g thymol. (**A**) The top twenty significantly enriched KEGG pathways associated with DEGs affected by 2.0 mg/g thymol exposure. (**B**) The top twenty significantly enriched KEGG pathways associated with DEGs affected by 4.0 mg/g thymol exposure. CK: The larvae were fed on a diet supplemented with DMSO; Thymol 2.0 and Thymol 4.0 denoted the larvae subjected to diets containing 2.0 and 4.0 mg/g of thymol, respectively.

KEGG pathway enrichment analysis was also performed on the thymol-responsive DEGs. For the 2.0 mg/g treatment group, 418 DEGs were annotated in the KEGG database. These genes were significantly enriched in 41 pathways (*p*.adjust < 0.05). The most prominent pathways were ‘carbon metabolism’ (27 DEGs), ‘purine metabolism’ (18 DEGs), and ‘biosynthesis of amino acids’ (17 DEGs). For the 4.0 mg/g treatment group, the DEGs were significantly enriched in 29 pathways. The most significantly enriched pathways included ‘ribosome’ (18 DEGs), ‘neuroactive ligand–receptor interaction’ (13 DEGs), and ‘ Peroxisome’ (11 DEGs). The top 20 significantly enriched KEGG pathways with the smallest *p*.adjust values affected by 2.0 and 4.0 mg/g thymol exposure are presented in Figure 5.

### 3.5. Classification of Thymol-Responsive Genes

GO enrichment analysis of DEGs found that numerous significantly enriched terms related to chitin metabolism and cuticle formation. Consequently, the DEGs associated with chitin metabolism were initially identified. In larvae exposed to 2.0 mg/g thymol, several key genes in chitin metabolism with differential expressions were identified (Supplemental Figure S1). These included genes encoding glucose-6-phosphate isomerase-like (G6PI), glutamine-fructose-6-phosphate aminotransferase 1-like (GFAT1), UDP-N-acetylhexosamine pyrophosphorylase-like protein 1 (UAP1), chitin synthase chs-2-like (CHS2), six chitinases (CHTs), β-N-acetylglucosaminidase 2 (NAG2), and four lytic polysaccharide monooxygenases (LPMOs). For the 4.0 mg/g thymol group, only four chitin metabolism-related genes were differentially expressed: *GFAT1, UAP1, CHT2*, and *NAG2*. The majority of the DEGs exhibited reduced expression following thymol exposure. In addition, the DEGs involved in cuticle assembly, specifically *chitin deacetylases* (*CDAs*) and *cuticle protein* genes, were further identified. In the 2.0 mg/g thymol treatment group, four *CDAs* were down-regulated. Furthermore, 68 *cuticle protein* genes were differentially expressed. All were down-regulated except for one gene encoding a larval cuticle protein LCP-22-like (LOC118282344). In the 4.0 mg/g thymol treatment group, 15 *cuticle protein* genes were DEGs, 14 of which were down-regulated. Our data suggest that thymol likely inhibits the growth of *S. frugiperda* by suppressing genes that are critical for chitin metabolism and cuticle formation.

The insect hormone biosynthesis pathway was significantly enriched with DEGs influenced by thymol exposure. Therefore, we performed additional analysis on the DEGs related to the JH III and 20-hydroxyecdysone (20E) biosynthesis pathways. A total of 16 DEGs were affected by 2.0 mg/g thymol exposure (Supplemental Figure S2). These DEGs encode aldehyde dehydrogenases (ALDHs), juvenile hormone epoxide hydrolases (JHEHs), juvenile hormone acid methyltransferase (JHAMT), cytochrome P450 315a1 (Shadow), glucose dehydrogenases (FADs), farnesoate epoxidase-like (CYP15A1_C1), cytochrome P450 307a1-like (Spook), and cytochrome P450 18a1-like. Among them, only three DEGs encoding JHAMT, JHEH, and cytochrome P450 18a1-like were down-regulated. Furthermore, twelve DEGs encoding ALDHs, JHEHs, FADs, cytochrome P450 15A1_C1, cholesterol 7-desaturase-like (NVD), and cytochrome P450 18a1-like were up-regulated in the larvae with 4.0 mg/g thymol treatment (Supplemental Figure S2). These findings indicate that thymol may modulate larval growth by influencing the hormone production pathway.

KEGG enrichment analysis identified DEGs affected by thymol exposure as being considerably enriched in two KEGG pathways: namely, fat digestion and absorption and protein digestion and absorption. Therefore, the DEGs producing lipases and proteases underwent additional analysis. A total of 20 *lipases* and 7 *phospholipases* were identified as DEGs influenced by 2.0 mg/g thymol exposure. Among them, five *lipases* and six *phospholipases* were down-regulated. Nine *lipases* with elevated expressions were identified in the larvae exposed to 4.0 mg/g thymol (Supplemental Figure S3A). Additionally, 17 *trypsins*, 6 *chymotrypsins*, 9 *carboxypeptidases*, 4 *aminopeptidases*, and 6 *serine proteases* were identified as DEGs influenced by 2.0 mg/g thymol exposure. Among them, four *trypsins*, three *carboxypeptidases*, one *aminopeptidase*, and six *serine proteases* were down-regulated. Furthermore, 13 *trypsins*, 3 *chymotrypsins*, 8 *carboxypeptidases*, 6 *aminopeptidases*, and 8 *serine proteases* were identified as DEGs influenced by 4.0 mg/g thymol exposure. Seven *trypsins*, two *carboxypeptidases*, and five *serine proteases* were down-regulated (Supplemental Figure S3B,C).

### 3.6. RT-qPCR Verification

**Figure 6:**
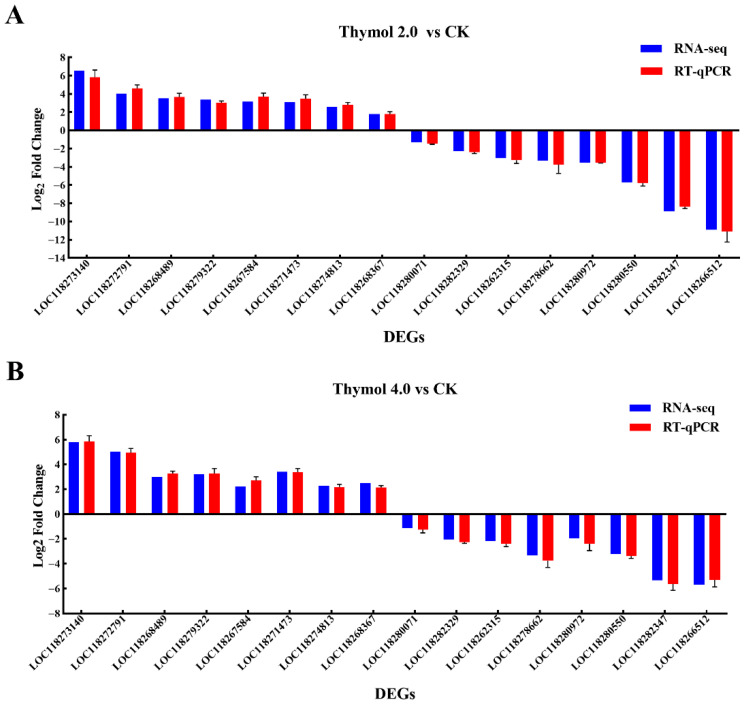
RT-qPCR validation of the chosen DEGs identified from larvae exposed to 2.0 and 4.0 mg/g thymol using RNA-Seq. (**A**) RT-qPCR validation results of the chosen DEGs identified from larvae exposed to 2.0 mg/g thymol. (**B**) RT-qPCR validation results of the chosen DEGs identified from larvae exposed to 4.0 mg/g thymol. Of these, 8 DEGs were up-regulated and 8 DEGs were down-regulated, respectively. The *y*-axis displayed the log_2_ fold change in each DEG. *Sf-EF1α* and *Sf-GAPDH* were used as references for normalization.

In this study, the expression profiles of sixteen DEGs affected by thymol treatments were further analyzed by RT-qPCR. As shown in Figure 6, the expression profiles of these DEGs determined by RT-qPCR corresponded with the trends observed in RNA-Seq data. These findings further validate the dependability of transcriptome data.

## 4. Discussion

Thymol has widely been identified as an effective alternative to conventional insecticides, demonstrating broad-spectrum efficacy against a diverse range of insect pests [22]. Studies have documented its insecticidal and growth-inhibitory actions against species including *Aphis craccivora*, *Pieris rapae*, *Spodoptera littoralis*, *Cimex lectularius*, *Spodoptera exigua*, and *H. armigera* [18,23,24,25,26]. Its repellent properties have also been noted against imported fire ants (*Solenopsis* spp.) [27]. These findings collectively indicate that thymol influences pests through multiple mechanisms. Our study further confirms the diverse bioactivity of thymol by demonstrating its insecticidal, growth-inhibiting, and development-disrupting effects on *S. frugiperda*. Prior studies indicated the insecticidal efficacy of thymol against third-instar *S. frugiperda* larvae (LD_50_ = 4.91 mg/g) [20]. Our findings further confirmed the insecticidal activity of thymol, as conducted by Lima et al. (2020) [20]. The apparent variations in insecticidal activity may be attributable to the sensitivity of distinct populations of *S. frugiperda*. Furthermore, comparative research indicates that carvacrol, the isomer of thymol, exhibits superior insecticidal efficacy against *S. frugiperda* larvae at tested dosages, suggesting both compounds as promising botanical alternatives [28]. Notably, thymol shows significant potential for integrated pest management through synergistic interactions. It has been shown to augment the toxicity of *Bacillus thuringiensis* crystals against lepidopteran pests [29]. It also enhanced the insecticidal effect of linalool against *Plutella xylostella* [30]. Similar synergistic effects with carvacrol have been documented in mosquito species [31,32]. These findings indicate a promising avenue for combining botanical compounds with conventional agents. Overall, thymol and its isomer carvacrol present a versatile foundation for alternative pest control strategies. The precise mechanisms underlying their toxicological diversity and the full potential of their synergistic combinations require further investigation.

High-throughput sequencing technology has facilitated the widespread use of RNA-Seq for investigating the mechanisms of action of efficient pest control agents. One RNA-Seq study indicated that carvacrol modulated the growth and development of *S. frugiperda* larvae by influencing the food digestion process [28]. Our prior research has shown that toosendanin altered gene expressions in the juvenile hormone and ecdysone signaling pathways, leading to the growth inhibition of *S. frugiperda* larvae [33]. This study further identified the DEGs influenced by thymol in the larvae of *S. frugiperda*. GO and KEGG enrichment analysis of DEGs indicated that thymol treatments could modulate chitin metabolism and cuticle synthesis, hormone biosynthesis, and food digestion. These disruptions collectively contribute to larval growth suppression and the mortality of *S. frugiperda* larvae. Our findings elucidate a preliminary mode of action of thymol against *S. frugiperda* larvae by RNA-Seq, thereby reinforcing the use of this technology in the toxicological mechanism analysis of insecticidal compounds. Moreover, the specific DEGs identified in this study may serve as targets for future research.

The insect cuticle is a complex and always renewing structure composed of chitin and cuticular proteins [34,35]. It serves as a load-bearing exoskeleton and protective outer barrier, safeguarding insects from predators and parasites, desiccation, and the penetration of foreign substances [36,37]. The growth and morphogenesis of insects are contingent upon the ability to modify the cuticle [38]. Chitin, a linear polymer of β-(1-4)-linked N-acetylglucosamines (GlcNAc), is the primary structural component of the cuticle. Its metabolism is tightly regulated by enzymes such as trehalases, chitin synthases, and chitinases during development [39,40]. Chitin metabolism is a well-established target for pesticides. For instance, validamycin disrupted gene expression related to chitin metabolism in *Bactrocera dorsalis*, leading to increased mortality and deformity [41]. Similarly, fenoxycarb impaired the growth of *Hyphantria cunea* by reducing the chitin content, elevating chitinase activity, and altering the expression of chitin-related genes [42]. This study revealed numerous genes associated with chitin metabolism as being DEGs influenced by thymol exposure. These findings indicate that disrupting chitin metabolism is a key mechanism of pest control. Thymol appears to exert its toxicity against *S. frugiperda* larvae by modifying the expression of essential genes within this pathway.

Cuticle proteins are another major component of the insect cuticle. They interact with each other and with chitin to form a stable framework, maintaining the elasticity and physical integrity of the exoskeleton [43]. Recent RNA interference (RNAi) studies suggest that altering cuticle protein expression often leads to developmental defects and malformations in insects, highlighting their potential as targets for pest control. For example, RNAi-mediated suppression of *HaLCP17* in *H. armigera* caused abnormalities in larval and pupal epidermis [44]. Silencing *ApCP7* and *ApCP62* in *Acyrthosiphon pisum* reduced the survival and reproductive rates [45]. In this study, nearly all DEGs encoding cuticle proteins exhibited down-regulated expression in larvae exposed to thymol. These findings indicate that thymol alters the expression of genes involved in both chitin metabolism and cuticle formation. Such disruptions likely disrupt cuticle renewal and molting processes, ultimately inhibiting the growth and development of *S. frugiperda* larvae. Therefore, the down-regulated cuticle protein genes and the DEGs associated with chitin metabolism may serve as prospective targets for thymol. Certainly, the particular mechanisms require additional investigation.

This study revealed that the insect hormone biosynthesis pathway was significantly enriched with DEGs influenced by thymol treatments. In the biosynthesis of juvenile hormone (JH III), retinal dehydrogenase (ALDH) is the principal enzyme responsible for synthesizing farnesoic acid [46]. Farnesal is converted to JH-III acid by farnesoate epoxidase CYP15A1_C1 [47]. Juvenile hormone epoxide hydrolase (JHEH) participates in the degradation of juvenile hormone II [46,47]. Our results showed that multiple DEGs related to juvenile hormone biosynthesis were up-regulated after thymol exposure. We speculate that thymol treatments might modify the expression of these DEGs to affect JH III levels. Ecdysone is a hormone that has a role in various physiological processes in insects. It is generated from cholesterol by a series of CYP450 enzymes, including CYP302A1 (Disembodied), CYP306A1 (Phantom), CYP307A1 (Spook), CYP314A1 (Shade), and CYP315A1 (Shadow) [47]. This study demonstrated that thymol treatments up-regulated *CYP307A1* (Spook) and *CYP315A1* (Shadow). Moreover, *CYP18A1*, a gene encoding an ecdysteroid 26-hydroxylase involved in 20E elimination, was down-regulated by 2.0 mg/g thymol but up-regulated by 4.0 mg/g thymol. The results indicate that the varying expression levels of *CYP18A1* may be associated with the differing quantities of thymol exposure. We speculate that thymol treatments may alter gene expressions to modulate the ecdysone titer. JH III and ecdysone jointly regulate insect molting, growth, and development through their precise concentrations and balance [48]. We hypothesize that thymol causes aberrant development in *S. frugiperda* larvae by modifying hormone biosynthesis gene expression, thus shifting the JH III and ecdysone levels and disrupting their equilibrium. Certainly, the particular mechanisms require additional investigation.

Insects thrive on Earth due to their remarkable capacity to extensively digest a diverse range of foods [49]. Nutrient digestion and absorption are critical physiological processes for their survival [50]. Our prior research demonstrated that carvacrol, an isomer of thymol, exhibited digestive toxicity against *S. frugiperda* by suppressing the activities of α-amylase, trypsin, and lipases and altering the expression of the corresponding genes [28]. The present study further demonstrates that thymol treatments greatly disrupt both protein and fat digestion and absorption pathways. These findings suggest that digestive toxicity may represent a common mechanism of action for the primary components in EOs derived from the Lamiaceae family. Specifically, the down-regulated genes encoding digestive enzymes could serve as potential molecular targets underlying thymol’s digestive toxicity. Conversely, the heightened expression of these DEGs indicates that *S. frugiperda* larvae may augment protein and fat digestion to generate increased energy in response to thymol poisoning. The precise function of these genes requires additional investigation.

## 5. Conclusions

In summary, the effects of thymol on the growth and survival of *S. frugiperda* were analyzed. In addition, 2.0 and 4.0 mg/g thymol treatments altered the development of *S. frugiperda*. RNA-Seq analysis of the survival larvae between the control group and the 2.0 and 4.0 mg/g thymol treatment groups were performed. GO and KEGG enrichment analyses found that the DEGs affected by thymol treatments were significantly enriched in several key biological pathways. These include chitin metabolism and cuticle synthesis, hormone biosynthesis, and protein digestion and absorption pathways. These findings provide preliminary insight into the toxicity and putative molecular mechanisms of thymol against *S. frugiperda* larvae, laying a preliminary foundation for its potential use in field-based pest control. It should be noted that the effects of thymol on *S. frugiperda* observed in this study were based on the data obtained from the laboratory. Thus, the actual field control effect of thymol requires further evaluation. In addition, due to the limitations of the transcriptome analysis, further functional verification of DEGs identified and analyzed in this study should be performed to fully elucidate the toxicological mechanism of thymol in *S. frugiperda*.

## Data Availability

The data presented in this study are available on request from the corresponding author due to privacy.

## Supplementary Materials

The following supporting information can be downloaded at: https://www.mdpi.com/article/10.3390/insects17010069/s1. Figure S1: Schematic depiction of the expression of DEGs associated with chitin metabolism and cuticle synthesis pathways; Figure S2: Schematic depiction of the expression of DEGs implicated in the JH III and 20-hydroxyecdysone (20E) biosynthesis pathways; Figure S3: Heatmaps depicting the expression of DEGs that encode enzymes associated with protein and fat digestion. Table S1: Primers used for RT-qPCR in the study; Table S2: Summary of the transcriptome data.

## References

[1] Shi Z., Li Y., Wu S., Xiao Y., Zeng W., Jia S., Xie Y., Yang Y., Tian L., Wang Y. (2024). The complete genome and biological activity of a novel *Spodoptera litura* multiple nucleopolyhedrovirus for controlling *Spodoptera frugiperda*. Biol. Control.

[2] Li T.H., Bueno A.F., Desneux N., Zhang L., Wang Z., Dong H., Wang S., Zang L.S. (2023). Current status of the biological control of the fall armyworm *Spodoptera frugiperda* by egg parasitoids. J. Pest Sci..

[3] Gouda M.N.R., Jeevan H., Shashank H.G. (2023). CRISPR/Cas9: A cutting-edge solution for combatting the fall armyworm, *Spodoptera frugiperda*. Mol. Biol. Rep..

[4] Montezano D.G., Sosa-Gómez D.R., Specht A., Roque-Specht V.F., Sousa-Silva J.C., Paula-Moraes S.D., Peterson J.A., Hunt T.E. (2018). Host plants of *Spodoptera frugiperda* (Lepidoptera: Noctuidae) in the Americas. Afr. Entomol..

[5] Lin Y., Huang Y., Liu J., Liu L., Cai X., Lin J., Shu B. (2023). Characterization of the physiological, histopathological, and gene expression alterations in *Spodoptera frugiperda* larval midguts affected by toosendanin exposure. Pestic. Biochem. Physiol..

[6] Song Y., Yang X., Li H., Wu K. (2023). The invasive *Spodoptera frugiperda* (J.E. Smith) has displaced *Ostrinia furnacalis* (Guenée) as the dominant maize pest in the border area of southwestern China. Pest Manag. Sci..

[7] van den Berg J., Britz C., du Plessis H. (2021). Maize yield response to chemical control of *Spodoptera frugiperda* at different plant growth stages in South Africa. Agriculture.

[8] Van den Berg J., du Plessis H. (2022). Chemical control and insecticide resistance in *Spodoptera frugiperda* (Lepidoptera: Noctuidae). J. Econ. Entomol..

[9] Wang H.H., Zhao R., Gao J., Zhang L., Zhang S., Liang P., Gao X., Gu S. (2023). Genetic architecture and insecticide resistance in Chinese populations of *Spodoptera frugiperda*. J. Pest Sci..

[10] Salerno G., Rebora M., Gorb S. (2023). Mechanoecology and chemoecology: Physical and chemical interactions between insects and plants. Insects.

[11] Pavela R., Guedes R.N.C., Maggi F., Desneux N., Benelli G. (2023). Essential oil antifeedants against armyworms: Promises and challenges. Entomol. Gen..

[12] Dervisoglou S., Traka C., Daferera D., Tarantilis P., Kakouri E., Kaparakou E., Revelou P.K., Polissiou M., Kavetsou E., Detsi A. (2023). Essential oils as a promising tool in the sustainable management of the tomato leafminer, *Tuta absoluta*: A review. Crop Prot..

[13] Usseglio V.L., Dambolena J.S., Zunino M.P. (2022). Can essential oils be a natural alternative for the control of *Spodoptera frugiperda*? A review of toxicity methods and their modes of action. Plants.

[14] Kachur K., Suntres Z. (2020). The antibacterial properties of phenolic isomers, carvacrol and thymol. Crit. Rev. Food Sci. Nutr..

[15] Kumrungsee N., Dunkhunthod B., Manoruang W., Koul O., Pluempanupat W., Kainoh Y., Yooboon T., Piyasaengthong N., Bullangpoti V., Nobsathian S. (2022). Synergistic interaction of thymol with *Piper ribesioides* (Piperales: Piperaceae) extracts and isolated active compounds for enhanced insecticidal activity against *Spodoptera exigua* (Lepidoptera: Noctuidae). Chem. Biol. Technol. Agric..

[16] Rojas A., Misic D., de Dicastillo C.L., Zizovic I., Velasquez E., Gutierrez D., Aguila G., Vidal C.P., Guarda A., Galotto M.J. (2023). A review on thymol-based bioactive materials for food packaging. Ind. Crops Prod..

[17] Marsin A.M., Muhamad I.I. (2023). Effectiveness of insect-repellent food packaging film incorporating thymol against rice weevil, *Sitophylus oryzae*. Curr. Sci..

[18] Bovornnanthadej T., Boonsoong B., Taylor D., Kainoh Y., Koul O., Bullangpoti V. (2013). Effect of thymol on reproductive biology of *Helicoverpa armigera* Hübner (Lepidoptera: Noctuidae). Commun. Agric. Appl. Biol. Sci..

[19] Yan T.K., Asari A., Salleh S.A., Azmi W.A. (2021). Eugenol and thymol derivatives as antifeedant agents against red palm weevil, *Rhynchophorus ferrugineus* (Coleoptera: Dryophthoridae) larvae. Insects.

[20] Lima A.P.S., Santana E.D.R., Santos A.C.C., Silva J.E., Ribeiro G.T., Pinheiro A.M., Santos I.T.B.F., Blank A.F., Araújo A.P.A., Bacci L. (2020). Insecticide activity of botanical compounds against *Spodoptera frugiperda* and selectivity to the predatory bug *Podisus nigrispinus*. Crop Prot..

[21] Shu B., Liu C., Huang Y., Lin Y., Zeng Y., Li S., Zeng J., Lin J., Zhang J. (2024). Cannibalism in *Spodoptera frugiperda* larvae: Effects of food lack, host plants, and food distribution. J. Asia-Pac. Entomol..

[22] Ruttanaphan T., Bullangpoti V. (2022). The potential use of thymol and (R)-(+)-pulegone as detoxifying enzyme inhibitors against *Spodoptera litura* (Lepidoptera: Noctuidae). Phytoparasitica.

[23] Tang X., Hou T. (2011). Isolation and identification of 2-isopropyl-5-methylphenol from *Stellera chamaejasme* and its insecticidal activity against *Aphis craccivora* and *Pieris rapae*. Nat. Prod. Res..

[24] Gaire S., Scharf M.E., Gondhalekar A.D. (2019). Toxicity and neurophysiological impacts of plant essential oil components on bed bugs (Cimicidae: Hemiptera). Sci. Rep..

[25] Tharamak S., Yooboon T., Pengsook A., Ratwatthananon A., Kumrungsee N., Bullangpoti V., Pluempanupat W. (2020). Synthesis of thymyl esters and their insecticidal activity against *Spodoptera litura* (Lepidoptera: Noctuidae). Pest Manag. Sci..

[26] Pengsook A., Tharamak S., Keosaeng K., Koul O., Bullangpoti V., Kumrungsee N., Pluempanupat W. (2022). Insecticidal and growth inhibitory effects of some thymol derivatives on the beet armyworm, *Spodoptera exigua* (Lepidoptera: Noctuidae) and their impact on detoxification enzymes. Pest Manag. Sci..

[27] Paudel P., Shah F.M., Guddeti D.K., Ali A., Chen J., Khan I.A., Li X.C. (2023). Repellency of carvacrol, thymol, and their acetates against imported fire ants. Insects.

[28] Liu J., Lin Y., Huang Y., Liu L., Cai X., Lin J., Shu B. (2023). The effects of carvacrol on development and gene expression profiles in *Spodoptera frugiperda*. Pestic. Biochem. Physiol..

[29] Konecka E., Czarniewska E., Kaznowski A., Grochowska J. (2018). Insecticidal activity of *Bacillus thuringiensis* crystals and thymol mixtures. Ind. Crops Prod..

[30] Webster A.E., Manning P., Sproule J.M., Faraone N., Cutler G.C. (2018). Insecticidal and synergistic activity of two monoterpenes against diamondback moth (Lepidoptera: Plutellidae). Can. Entomol..

[31] Silva V.B., Travassos D.L., Nepel A., Barison A., Costa E.V., Scotti L., Scotti M.T., Mendonça-Junior F.J.B., La Corte Dos Santos R., de Holanda Cavalcanti S.C. (2017). Synthesis and chemometrics of thymol and carvacrol derivatives as larvicides against *Aedes aegypti*. J. Arthropod Borne Dis..

[32] Youssefi M.R., Tabari M.A., Esfandiari A., Kazemi S., Moghadamnia A.A., Sut S., Dall’Acqua S., Benelli G., Maggi F. (2019). Efficacy of two monoterpenoids, carvacrol and thymol, and their combinations against eggs and larvae of the west nile vector *Culex pipiens*. Molecules.

[33] Shu B., Lin Y., Huang Y., Liu L., Cai X., Lin J., Zhang J. (2024). Characterization and transcriptomic analyses of the toxicity induced by toosendanin in *Spodoptera frugipreda*. Gene.

[34] Charles J.P. (2010). The regulation of expression of insect cuticle protein genes. Insect Biochem. Mol. Biol..

[35] Ren Y., Li Y., Ju Y., Zhang W., Wang Y. (2023). Insect cuticle and insecticide development. Arch. Insect Biochem. Physiol..

[36] Qi H., Liu T. (2025). Cuticular proteins: Essential molecular code for insect survival. Insect Biochem. Mol. Biol..

[37] Zhang C.X., Moussian B. (2025). Mini-review: Aspects of cuticle formation and structure advanced by studies in *Nilaparvata lugens*. Insect Biochem. Mol. Biol..

[38] Merzendorfer H., Zimoch L. (2003). Chitin metabolism in insects: Structure, function and regulation of chitin synthases and chitinases. J. Exp. Biol..

[39] Yu A., Beck M., Merzendorfer H., Yang Q. (2024). Advances in understanding insect chitin biosynthesis. Insect Biochem. Mol. Biol..

[40] Liu X.J., Liu W.M., Zhao X.M., Zhang J.Z., Ma E.B. (2019). Progress in the study of insect cuticle development and prospects for future research. Chin. J. Appl. Entomol..

[41] Li Y., Xu Y., Wu S., Wang B., Li Y., Liu Y., Wang J. (2023). Validamycin inhibits the synthesis and metabolism of trehalose and chitin in the oriental fruit fly, *Bactrocera dorsalis* (Hendel). Insects.

[42] Zou H., Gao Y., Zhang S., Liu T., Zhang G. (2025). Regulation of chitin synthesis by the juvenile hormone analogue fenoxycarb in *Hyphantria cunea*. Pestic. Biochem. Physiol..

[43] Chen E.H., Hou Q.L. (2021). Identification and expression analysis of cuticular protein genes in the diamondback moth, *Plutella xylostella* (Lepidoptera: Plutellidae). Pestic. Biochem. Physiol..

[44] Zhang M.K., Wang F.F., Qin P., Chen J., Huang Y.Y., Yu L., Meng J.Y., Sang W. (2024). Imidazole-modified graphene quantum dots can effectively promote the efficient silencing of the larval cuticle protein gene *HaLCP17* in *Helicoverpa armigera*. Entomol. Gen..

[45] Ma R., Wu Y., Liu H., Sun Q., Song L., Liu L., Wang S., Dewer Y. (2024). The cuticular protein gene *ApCP7* and *ApCP62* are essential for reproduction in *Acyrthosiphon pisum*, affecting ecdysis and survival. Int. J. Biol. Macromol..

[46] Shu B., Yu H., Li Y., Zhong H., Li X., Cao L., Lin J. (2021). Identification of azadirachtin responsive genes in *Spodoptera frugiperda* larvae based on RNA-seq. Pestic. Biochem. Physiol..

[47] Jia Z.Q., Zhan E.L., Zhang S.G., Jones A.K., Zhu L., Wang Y.N., Huang Q.T., Han Z.J., Zhao C.Q. (2022). Sublethal doses of broflanilide prevents molting in the fall armyworm, *Spodoptera frugiperda* via altering molting hormone biosynthesis. Pestic. Biochem. Physiol..

[48] Wang P., Cui Q., Wang X., Liu Y., Zhang Y., Huang X., Jiang S., Jiang M., Bi L., Li B. (2023). The inhibition of ecdysone signal pathway was the key of pyriproxyfen poisoning for silkworm, *Bombyx mori*. Pestic. Biochem. Physiol..

[49] Holtof M., Lenaerts C., Cullen D., Vanden Broeck J. (2019). Extracellular nutrient digestion and absorption in the insect gut. Cell Tissue Res..

[50] Li X.Y., Si F.L., Zhang X.X., Zhang Y.J., Chen B. (2024). Characteristics of trypsin genes and their roles in insecticide resistance based on omics and functional analyses in the malaria vector *Anopheles sinensis*. Pestic. Biochem. Physiol..

