# Supplementary material for: Growth Inhibition, Mortality Induction, Adverse Impacts of Development, and Underlying Molecular Mechanisms of Thymol Against Spodoptera frugiperda
Source: Insects. 2026 Jan 6;17(1):69. doi: 10.3390/insects17010069 (PMC12841978; doi:10.3390/insects17010069)
Supplement: Supplementary file 1 [file insects-17-00069-s001.zip › insects-4020796-supplementary.pdf]

Growth Inhibition, Mortality Induction, Adverse Impacts of Development, and Underlying Molecular Mechanisms of Thymol Against *Spodoptera frugiperda*

Huiyin Hu [1,†], Huanqian Yao [1,†], Shuyin He [1], Xinyi Xie [1], Cuiting Liu [1], Veeran Sethuraman [2], Jingjing Zhang [1,*] and Benshui Shu [1,*]

[1] Guangzhou City Key Laboratory of Subtropical Fruit Trees Outbreak Control, Zhongkai University of Agriculture and Engineering, Guangzhou 510225, China

[2] Department of Orthopaedics, Saveetha Medical College and Hospital, Saveetha Institute of Medical and Technical Sciences (SIMATS), Chennai 602105, Tamil Nadu, India

* Correspondence: zjjscau@scau.edu.cn (J.Z.); shubenshui@126.com (B.S.)

† These authors contributed equally to this work.

Supplement Table S1. Primers used for RT-qPCR in the study

Primer name	Primer Sequence (5'-3')	Primer name	Primer Sequence (5'-3')
LOC118272791-RT-F	ATGGGAGGATGGAACGTAGAA	LOC118272791-RT-R	AAGTCGTAAGGCGGAGGTGT
LOC118273140-RT-F	AAACTTTGTCACCTGGCTCATC	LOC118273140-RT-R	CGTTGCTCGGGCTTAATCTA
LOC118279322-RT-F	CCTGAAGACATTCTCGGACACC	LOC118279322-RT-R	GGGAAGAAGGCACCAACGA
LOC118267584-RT-F	TGGCAATGACTTCTGGGTTTT	LOC118267584-RT-R	AGTGAGCAGCGGTAAGGACG
LOC118274813-RT-F	GGCTACTTGCAGAAACAACTACG	LOC118274813-RT-R	CCAATACCGAAAGAGCAGACAC
LOC118271473-RT-F	TTCACCGACGACCGAATGT	LOC118271473-RT-R	ATAGCCGTGCGAGCCAAAT
LOC118268367-RT-F	TCGCTTTGATGAGGTCCGA	LOC118268367-RT-R	CAACCAGTTGAAACCGCTGA
LOC118268489-RT-F	GCTAAAGTCGCTGAGGGTGAT	LOC118268489-RT-R	AATGTTTTGGCGTGGGCTA
LOC118262315-RT-F	GGTCCGCTGGCTTTAATCG	LOC118262315-RT-R	CCTTGGAGGCAGTCTACTGTTTT
LOC118280972-RT-F	GCATCTCCACGCTCATTCC	LOC118280972-RT-R	CTGCTTTCTGCGGCTTCAT
LOC118280071-RT-F	ACATACTCCAACTTCGCCTACCC	LOC118280071-RT-R	GCATTACCTCCTTTGTCCTCCA
LOC118282347-RT-F	CAGCTACAGCTACGCTCTTGAA	LOC118282347-RT-R	GGAGACGACTGGGACTATTGG
LOC118280550-RT-F	CTTGGTCATCTACAGCCGAAAA	LOC118280550-RT-R	CGGTGGGTCCATTCAGGTT
LOC118278662-RT-F	AGGGATACTTGCGAGGGTGA	LOC118278662-RT-R	TACTGCCGAGGATGCCACA
LOC118282329-RT-F	GTGAAGCTCTGCTGGCGTAC	LOC118282329-RT-R	GGCGGATAGGTCTTGGGAT
LOC118266512-RT-F	CAGCCTACCCTGAACAATCCA	LOC118266512-RT-R	CCGTTCTCAGTTTCGTAGTTGTATC
RPL13-RT-F	GCCTTAACCCTGCTTTTGCTAG	*RPL13*-RT-R	GCTTCGCCCTTCAATACCTTC
EF1α-RT-F	TGGGCGTCAACAAAATGGA	*EF1α*-RT-R	TCTCCGTGCCAGCCAGAAAT

Supplemental Table S2 Summary of the transcriptome data

Sample	Raw reads	Clean reads	Clean data (bp)	Q20 (%)	Q30 (%)	GC (%)
CK1	48,784,270	48,480,640	6,765,762,609	98.98	96.70	47.82
CK2	45,058,006	44,770,226	6,206,767,528	98.83	96.24	48.99
CK3	43,712,302	43,469,258	5,999,350,366	99.01	96.78	48.19
Thymol 2.0-1	48,672,998	48,410,548	6,686,476,546	98.89	96.80	47.64
Thymol 2.0-2	54,794,800	54,457,584	7,618,939,736	99.01	96.38	46.28
Thymol 2.0-3	48,880,206	48,607,584	6,776,520,888	98.84	96.77	47.14
Thymol 4.0-1	54,924,954	54,601,630	7,434,554,931	98.81	96.20	48.24
Thymol 4.0-2	42,574,594	42,301,054	5,786,735,916	98.85	96.28	27.56
Thymol 4.0-3	40,442,464	40,227,176	5,365,926,403	99.02	96.88	43.52

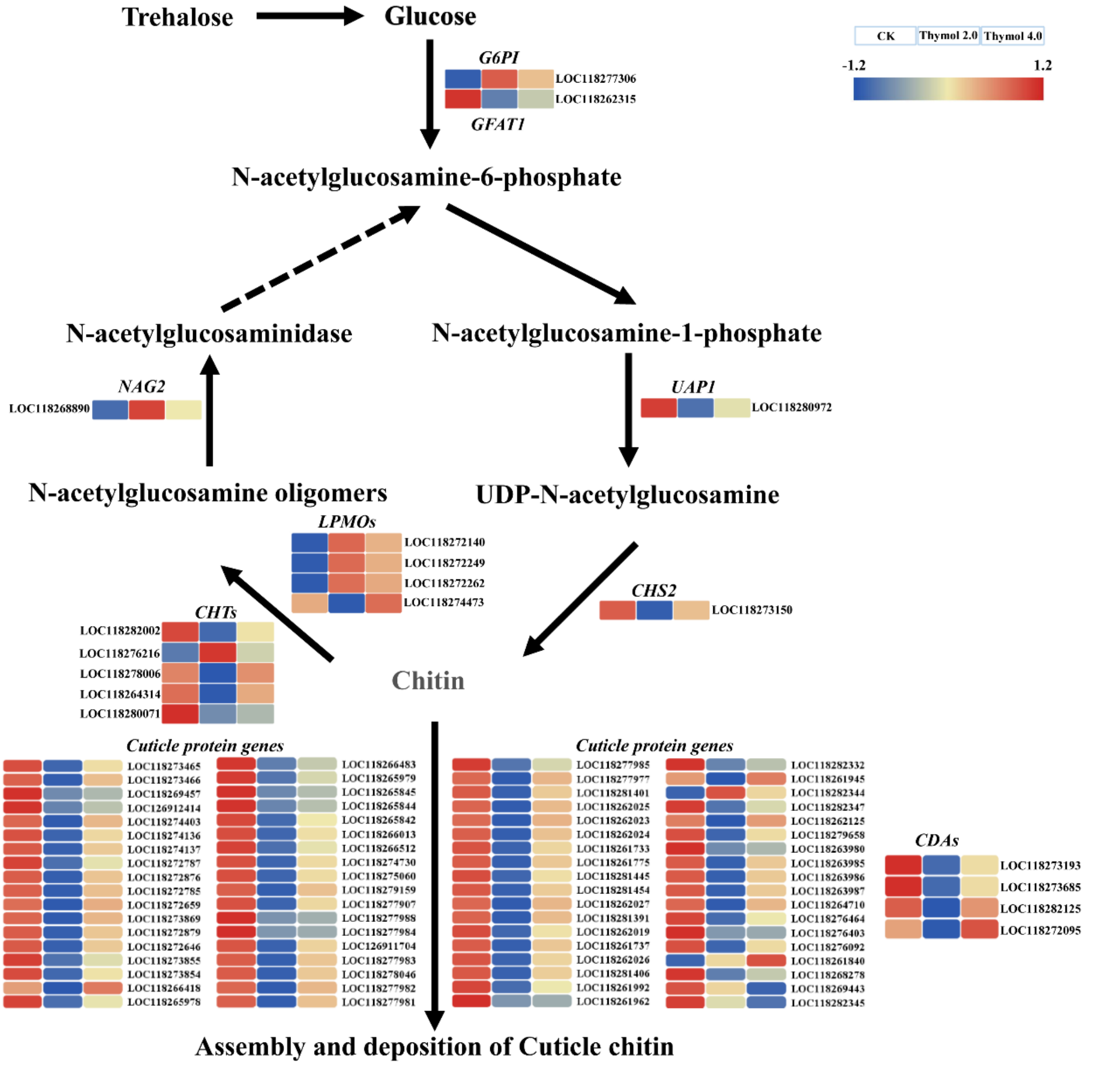

Supplemental Figure S1. Schematic depiction of the expression of DEGs associated with chitin metabolism and cuticle synthesis pathways. CK: The larvae were fed on a diet supplemented with DMSO; Thymol 2.0 and Thymol 4.0 denoted the larvae subjected to diets containing 2.0 and 4.0 mg/g of thymol, respectively. The arrows represented the biological process by which one substance is transformed into another. Heatmaps in blue and red signify lower and higher expression levels, respectively.

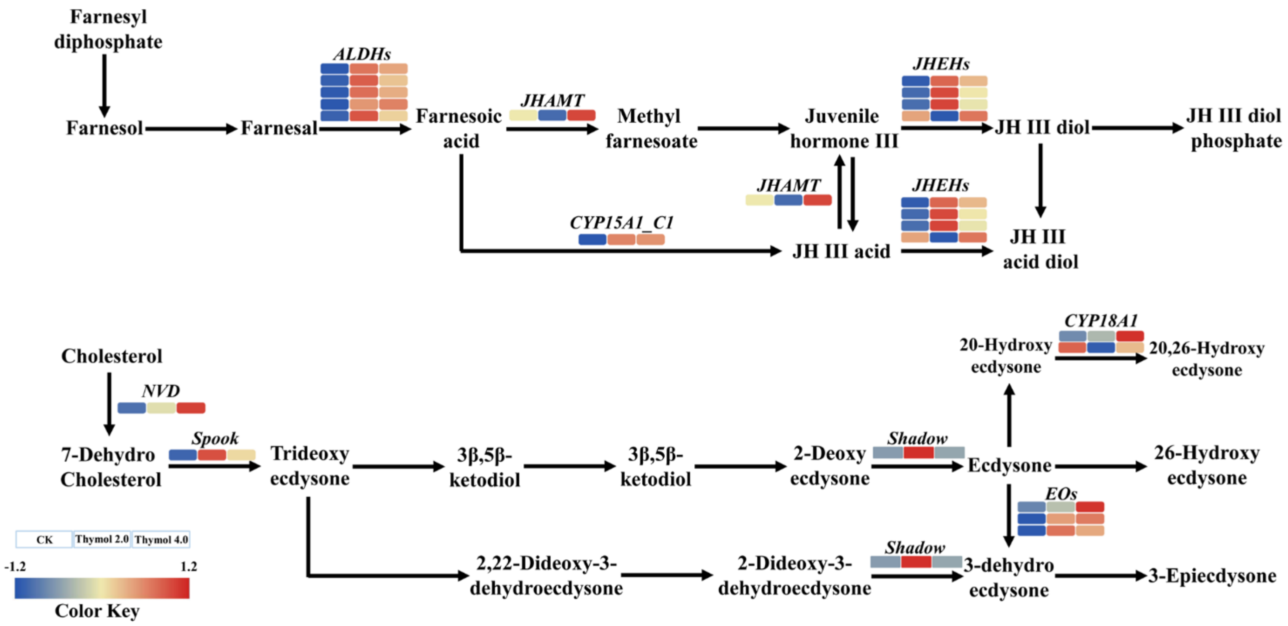

Supplemental Figure S2. Schematic depiction of the expression of DEGs implicated in the JH III and 20-hydroxyecdysone (20E) biosynthesis pathways. CK: The larvae were fed on a diet supplemented with DMSO; Thymol 2.0 and Thymol 4.0 denoted the larvae subjected to diets containing 2.0 and 4.0 mg/g of thymol, respectively. The arrows represented the biological process by which one substance is transformed into another. Heatmaps in blue and red signify lower and higher expression levels, respectively.

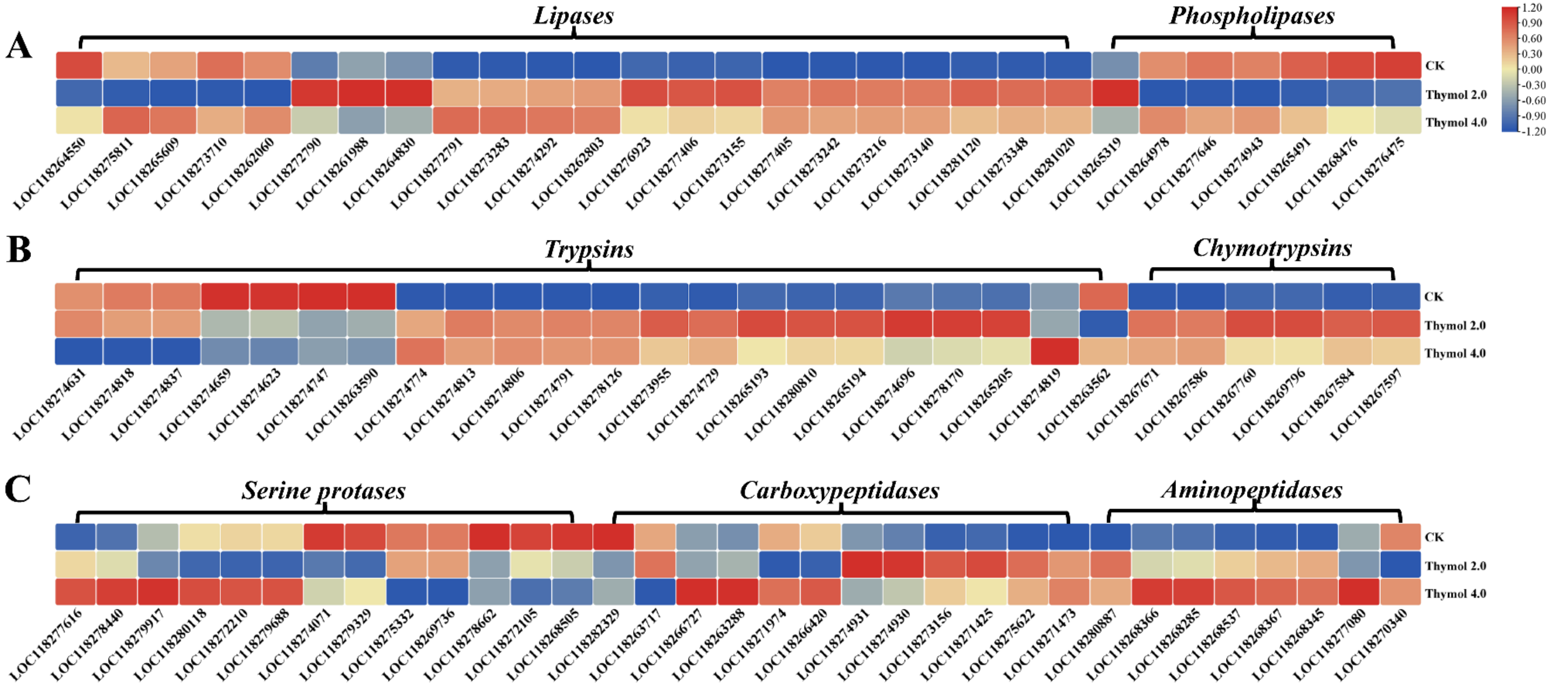

Supplemental Figure S3. Heatmaps depicting the expression of DEGs that encode enzymes associated with protein and fat digestion. A: The heatmap representations of DEGs encoding lipases and phospholipases influenced by thymol exposure. B: The heatmap representations of DEGs encoding trypsins and chymotrypsins influenced by thymol exposure. C: The heatmap representations of DEGs encoding serine proteases, carboxypeptidases, and aminopeptidases influenced by thymol exposure. CK: The larvae were fed on a diet supplemented with DMSO; Thymol 2.0 and Thymol 4.0 denoted the larvae subjected to diets containing 2.0 and 4.0 mg/g of thymol, respectively. Heatmaps in blue and red signify lower and higher expression levels, respectively.
